# Thrombospondin1 Deficiency Reduces Obesity-Associated Inflammation and Improves Insulin Sensitivity in a Diet-Induced Obese Mouse Model

**DOI:** 10.1371/journal.pone.0026656

**Published:** 2011-10-24

**Authors:** Yanzhang Li, Xiaopeng Tong, Courtney Rumala, Kate Clemons, Shuxia Wang

**Affiliations:** Graduate Center for Nutritional Sciences, University of Kentucky, Lexington, Kentucky, United States of America; The University of Hong Kong, Hong Kong

## Abstract

**Background:**

Obesity is prevalent worldwide and is associated with insulin resistance. Advanced studies suggest that obesity-associated low-grade chronic inflammation contributes to the development of insulin resistance and other metabolic complications. Thrombospondin 1 (TSP1) is a multifunctional extracellular matrix protein that is up-regulated in inflamed adipose tissue. A recent study suggests a positive correlation of TSP1 with obesity, adipose inflammation, and insulin resistance. However, the direct effect of TSP1 on obesity and insulin resistance is not known. Therefore, we investigated the role of TSP1 in mediating obesity-associated inflammation and insulin resistance by using TSP1 knockout mice.

**Methodology/Principal Findings:**

Male TSP1-/- mice and wild type littermate controls were fed a low-fat (LF) or a high-fat (HF) diet for 16 weeks. Throughout the study, body weight and fat mass increased similarly between the TSP1-/- mice and WT mice under HF feeding conditions, suggesting that TSP1 deficiency does not affect the development of obesity. However, obese TSP1-/- mice had improved glucose tolerance and increased insulin sensitivity compared to the obese wild type mice. Macrophage accumulation and inflammatory cytokine expression in adipose tissue were reduced in obese TSP1-/- mice. Consistent with the local decrease in pro-inflammatory cytokine levels, systemic inflammation was also decreased in the obese TSP1-/- mice. Furthermore, in vitro data demonstrated that TSP1 deficient macrophages had decreased mobility and a reduced inflammatory phenotype.

**Conclusion:**

TSP1 deficiency did not affect the development of high-fat diet induced obesity. However, TSP1 deficiency reduced macrophage accumulation in adipose tissue and protected against obesity related inflammation and insulin resistance. Our data demonstrate that TSP1 may play an important role in regulating macrophage function and mediating obesity-induced inflammation and insulin resistance. These data suggest that TSP1 may serve as a potential therapeutic target to improve the inflammatory and metabolic complications of obesity.

## Introduction

The worldwide obesity epidemic is a major risk factor for type 2 diabetes and cardiovascular disease. Obesity is now recognized as a state of chronic low-grade systemic inflammation which promotes the development of insulin resistance and other metabolic complications [1]. Obesity is associated with macrophage infiltration into adipose tissue and the dysregulated production of adipokines [2]. Adipose tissue macrophages (ATMs) are the primary source of inflammatory cytokine production in adipose tissue and play a key role in obesity-induced chronic low-grade inflammation and insulin resistance [2]. Although there have been some advances in the study of ATMs in obese conditions [2], [3], [4], the mechanisms underlying inflammatory cell recruitment and activation are not completely understood.

Thrombospondin1 (TSP1) is a major component of platelet alpha granules [5], [6]. TSP1 acts as an immediate early response gene, exhibiting rapid but transient induction by growth factors and stress in many cell types including adipocytes and macrophages [7], [8], [9], [10]. TSP1 exists as both a component of the extracellular matrix and as a soluble molecule found in various body fluids and in the cell culture conditioned medium. TSP1 is a 420–450 kDa homotrimer with individual subunits of approximately 145 kDa. The diverse biological activities of TSP1 have been mapped to specific domains of the molecule by interaction with different cell surface receptors [11], [12], [13], [14], [15], [16]. TSP1 is a major regulator of latent TGF-β activation, a well-known endogenous angiogenesis inhibitor, and a regulator of cell proliferation and adhesion [9], [11], [17], [18], [19], [20], [21]. TSP1 also plays a role in inflammation and obesity. TSP1 has been shown to be expressed in visceral adipose tissue of rats and humans [22], [23]. Its expression is markedly regulated during the differentiation of preadipocytes into mature adipocytes [24], [25]. TSP1 is up-regulated in developing adipose tissue of mice with diet or genetically induced obesity [26]. In obese, insulin resistant humans, TSP1 was recently reported to be up-regulated and associated with adipose inflammation and insulin resistance [27]. However, in vivo studies examining the role of TSP1 in regulating macrophage function and obesity-associated inflammation and insulin resistance are lacking.

In the current study, we utilized TSP1 knockout and wild type mice to investigate the role of TSP1 in high fat diet induced obesity, inflammation, and insulin resistance. Using this diet-induced obesity paradigm, we demonstrated that TSP1 deletion had no effect on obesity development. However, these obese TSP1 deficient mice had improved glucose tolerance and insulin sensitivity. This improved glucose-insulin homeostasis was found to be associated with significantly decreased macrophage accumulation and inflammation in the adipose tissue. In vitro studies further supported the effect of TSP1 on macrophage mobility and function. Together, these data demonstrate that TSP1 may play an important role in obesity-associated insulin resistance partially through regulating macrophage function and inflammation.

## Materials and Methods

### Ethics Statement

All experiments involving mice conformed to the National Institutes of Health Guide for the Care and Use of Laboratory Animals and were approved by the University of Kentucky Institutional Animal Care and Use Committee. This protocol was approved under application number 00966M2005.

### Experimental animals and protocols

Eight week old male TSP1-/- mice (on C57BL6/J background, purchased from Jackson Laboratory) and age-matched littermate controls were used in the study. Mice were housed in a temperature controlled room with a 12 hour light/dark cycle. Mice were fed a LF (10% kcal as fat; D12450B; research Diets, Inc, NJ) or a HF diet (60% kcal as fat; D12492, research Diet, Inc, NJ) for 16 weeks. Each group contained 10–15 mice.

### Metabolic measurements

The blood was collected from animals after 6 hr fasting. Plasma glucose levels were measured using a Glucometer. Plasma total cholesterol and triglyceride levels were measured using kits from Wako Chemicals. Plasma insulin, leptin, IL-6, MCP-1, TNF-α, PAI-1, resistin and adiponectin concentrations were measured using a mouse adipokine assay kit (Millipore).

### Glucose tolerance and Insulin sensitivity tests

After 15 weeks of LF and HF-feeding, glucose tolerance was analyzed in animals after 6 h fasting. Following an intraperitoneal injection of glucose (1 g/kg body weight), blood glucose concentrations were measured using a Glucometer at 0, 15, 30, 60, and 120 minutes after injection. For insulin sensitivity assessment, Insulin (0.5 unit/kg body weight) (Novolin R, Novo Nordisk InC.) was injected into mice intraperitonealy. Similarly, blood glucose levels were measured at 0, 15, 30, 60, and 120 minutes after injection to assess insulin's effect.

### Assessments of body composition, food intake and energy expenditure

At 15 weeks, mice were put in TSE LabMaster chambers (TSE systems) individually for 5 days for measurement of food intake, water intake and indirect calorimetry. In addition, EchoMRI (Echo Medical System) was used to evaluate body fat and lean content in mice after 16 weeks of LF or HF feeding.

### Real-time PCR

Total RNA was isolated from epididymal fat tissue of TSP1-/- and wild type control mice using TRIZOL reagent (Invitrogen, Carlsband, CA) and treated with DNaseI (Roche, Indianapolis, IN). The treated RNA was cleaned up using RNeasy kit (Qiagen, Valencia, CA). Total RNA of 2 µg was used for cDNA synthesis using High Capacity cDNA Reverse Transcription Kit (Invitrogen, Carlsband, CA). Real-time PCR analyses were performed using SYBR Green PCR Master Mix kit with a MyiQ Real-time PCR Thermal Cycler (Bio-Rad). All reactions were performed in triplicate in a final volume of 25 µl. Dissociation curves were run to detect nonspecific amplification and we confirmed that single products were amplified in each reaction. The quantities of each test gene and internal control 18S RNA were then determined from the standard curve using the MyiQ system software and mRNA expression levels of test genes were normalized to 18S RNA levels. The primer sequences are shown in **Table 1**.

**Table 1:** Sequences of primers used in the study.

Genes	Forward (5′-3′)	Reverse (5′-3′)
18 sRNA	AGTCGGCATCGTTTATGGTC	CGAAAGCATTTGCCAAGAAT
IL-6	CTG CAAGAGACTTCCATCCAGTT	GAAGTAGGGAAGGCCGTGG
TNF-α	AGCCGATGGGTTGTACCT	TGAGTTGGTCCCCCTTCT
MCP-1	CAGCCAGATGCAGTTAACGC	GCCTACTCATTGGGATCATCTTG
F4/80	CTTTGGCTATGGGCTTCCAGTC	GCAAGGAGGACAGAGTTTATCGTG
CD11c	CTGGATAGCCTTTCTTCTGCTG	GCACACTGTGTCCGAACTC
TGF-β1	ACTGCTTCCCGAATGTCTGACGTA	TAAAGAGGTCACCCGCGTGCTAAT
PAI-1	GCG TGTCAGCTCGTCTACAG	GTACTGCGGATGCCATCTTT
iNOS	CCAAGCCCTCACCTACTACTTCC	CTCTGAGGGCTGACACAAGG

### Immunohistochemical staining

Epididymal adipose tissue was fixed and embedded in paraffin. Paraffin fixed adipose tissues were cut into 4–5 µm sections and placed onto slides. Sections were deparaffinized in xylene, and were rehydrated in graded mixtures of ethanol/water. Endogenous peroxidase activity was blocked with 3% H_2_O_2_ for 30 min at room temperature (RT). The slides were placed in PBS buffer containing 5% BSA for 30 min. A rat anti-mouse F4/80 antibody (AbD Serotec, Raleigh, NC) was applied for 1 hour at RT. A negative control was included by substituting control IgG for the primary antibody. After washing with PBS, biotinylated secondary antibody was applied for 30 min. After another 15 min washing, an avidin-biotin-peroxidase complex was applied to the slides for 30 min. The slides were washed once again with PBS before color development with DAB using Vectastain ABC system (Vector Lab).

### Macrophage function studies

#### Studies using bone marrow derived cells

Bone-marrow derived cells were isolated from femurs and tibias of male WT and TSP1-/- mice as described previously [3]. For seven days, these cells were cultured in RPMI-1640 media containing 20% FBS, 25 ng/ml M-CSF (Sigma), and penicillin/streptomycin to allow proliferation and differentiation into mature macrophages. Macrophages were then plated and treated with or without lipopolysaccaride (LPS: 100 ng/ml) for 3 hr. After treatment, cells were harvested and expression of proinflammatory cytokines was determined by real-time PCR.

#### Macrophage migration and adhesion assay

Mice (male TSP1-/- , CD36 -/-, and wild type control mice) were sacrificed and macrophages were harvested by lavage of the peritoneal cavity with sterile PBS [28]. The cells were washed once with serum-free DMEM media, counted and used immediately in a migration or cell adhesion assay. For migration assay: Peritoneal macrophages (1×10^6^) from male WT mice or TSP1-/- mice were loaded into the upper chambers, while the lower chambers were filled with DMEM media containing either purified TSP1 (5 µg/ml, from R&D system) or MCP1 (50 ng/ml). Transwell plates were then incubated at 37°C for 5 hours. Media was removed from the upper chamber, and the cells in the bottom chamber were then fixed in methanol and stained with Giemsa solution (Dade Behring, Marburg, Germany). Cell counts were performed by two different observers who were blinded to the study design.

#### For adhesion assay

To assess cell spreading, macrophages were plated into four-chambered LAB-TEK slides (Nalge Nunc International; Naperville, IL) uncoated or precoated with purified TSP1 or fibronectin for 6 hours. The cells were then washed with PBS, fixed with 4% par formaldehyde, permeabilized with 0.1% Trixton X-100, and blocked with 1% BSA for 30 min before staining with Alexa-Fluo 568-conjugated or FITC conjugated phalloidin (molecular Probes, Eugene, OR). The slides were mounted in prolong anti-fade reagent (Molecular Probes). Random images of at least 25 cells from three or more independent experiments were digitally captured using a Leica TCS SP confocal microscope (UK imaging center). Individual cells were outlined and total cell area was quantified using Metamorph software.

### Statistical analysis

Data are the mean±SE. Differences between groups were determined by ANOVA followed by Turkey's post hoc tests or Student's t-test as appropriate. The significance level was p<0.05.

## Results

### TSP1 deficiency does not affect the development of diet induced obesity

**Figure 1:**
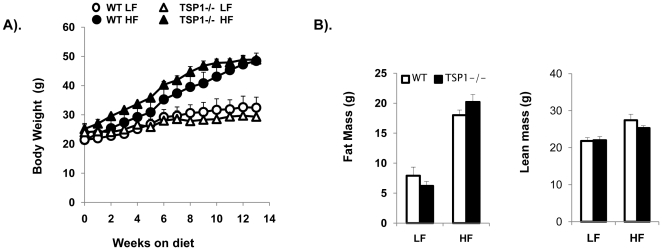
TSP1 deficiency had no effect on obesity development in a high fat diet induced obese mouse model. Male TSP1-/- mice and wild type littermate controls were fed LF or HF diet for 16 weeks. **A):** Graphs showing the increase of body weight over time on diets. **B):** Fat and lean mass of different groups of animals. Data are represented as mean±SE (n = 10 mice/group).

To determine whether TSP1 deficiency affects the development of obesity, male TSP1-/- mice and wild type controls were fed with a low fat (LF, 10% fat) or high fat (HF, 60% fat) diet for 16 weeks. The body weight was measured weekly. Prior to the end of the study, body composition was analyzed using EchoMRI. The results showed that body weight and fat mass were similar between the TSP1-/- and wild type control mice under LF or HF feeding conditions (**Figure 1**). In addition, high fat feeding significantly increased plasma triglyceride levels in WT and TSP1-/- mice. However, plasma triglyceride levels were less in HF-fed TSP1-/- mice than those in HF-fed WT mice (**Table 2**). Total cholesterol levels were similarly increased in both HF-fed WT and TSP1-/- mice (**Table 2**). We also measured metabolic parameters such as food intake, oxygen consumption and physical activity, and did not observe a difference between HF-fed WT mice and HF-fed TSP1-/- mice (data not shown). Together, these data suggest that TSP1 deficiency does not affect the development of obesity.

**Table 2:** Metabolic parameters of LF and HF feeding mice.

Parameters	WT (LF)	TSP1-/-(LF)	WT (HF)	TSP1-/- (HF)
Glucose(mg/dl)	131±9.3	122±9.5	163±12*	168±7.8∧
Insulin (ng/ml)	0.499±0.03	0.427±0.02	3.64±1.13*	1.72±0.25∧ #
TG(mg/dl)	15.5±0.7	14.03±2.32	34.13±1.96*	24.58±1.9∧ #
TC (mg/dl)	102.1±2.7	77.1±6.9	151.5±26.2	140.6±14.5∧
NEFA (mEq/L)	1.06±0.1	0.78±1.08	0.79±0.13	0.64±0.02

TG: triglycerides; TC: total cholesterol. Data are means±SE; n = 6 mice/group.

*P<0.05 vs. WT (LF).

∧ P<0.05 vs. TSP1-/- LF.

# P<0.05 vs. WT (HF).

### TSP1-/- mice exhibit improved glucose tolerance and increased insulin sensitivity as compared to WT controls under HF feeding conditions

**Figure 2:**
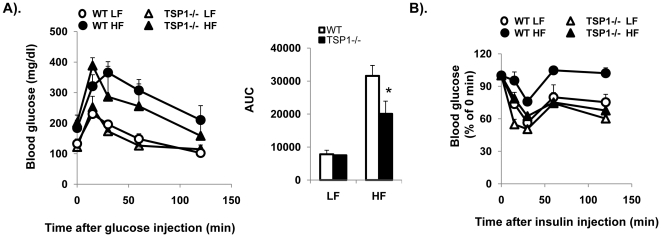
Obese TSP1-/- mice had improved glucose tolerance and insulin sensitivity. Male TSP1-/- mice and wild type littermate controls were fed LF or HF diet for 16 weeks. Intraperitoneal glucose tolerance (A) and insulin sensitivity test (B) were measured. Changes in blood glucose levels were monitored over time. Data are represented as mean±SE (n = 10 mice/group). *P<0.05 vs. WT HF group. AUC: area under the curve.

Recent studies suggest that adipose TSP1 levels are inversely associated with insulin sensitivity in obese subjects [27]. Although our data indicates that TSP1 deficiency does not affect the development of obesity, it is not known whether TSP1 deficiency affects obesity associated insulin resistance. Therefore, fasting blood glucose and insulin levels were measured. Glucose tolerance test (GTT) and insulin sensitivity assay (ITT) were performed in LF and HF fed mice. The results showed that fasting blood glucose levels were similarly increased in both HF feeding TSP1-/- and wild type control mice. HF feeding also increased the insulin levels in both genotypes. However, the insulin was increased to a significantly lower extent in TSP1-/- mice (**Table 2**). Furthermore, GTT and ITT tests demonstrated that HF-fed TSP1-/- mice had improved glucose tolerance (**Figure 2A**) and insulin sensitivity (**Figure 2B**).

### HF-fed TSP1-/- mice have decreased macrophage accumulation in adipose tissue and decreased inflammation

**Figure 3:**
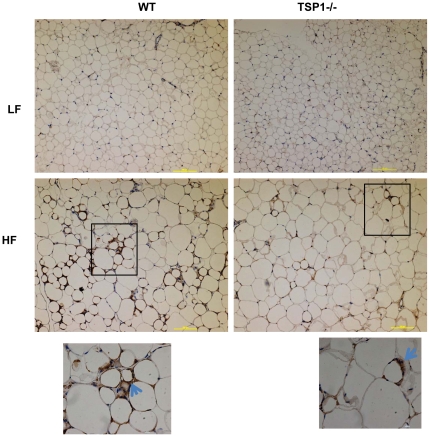
Obese TSP1-/- mice had decreased macrophage accumulation in adipose tissue. Representative images of epididymal fat tissue from four groups of mice stained with anti F4/80 antibody to identify macrophage accumulation in fat tissue. The blue arrow head indicates a crown like structure. The positive staining showed brown color. The scale bar represents 100 µm.

**Figure 4:**
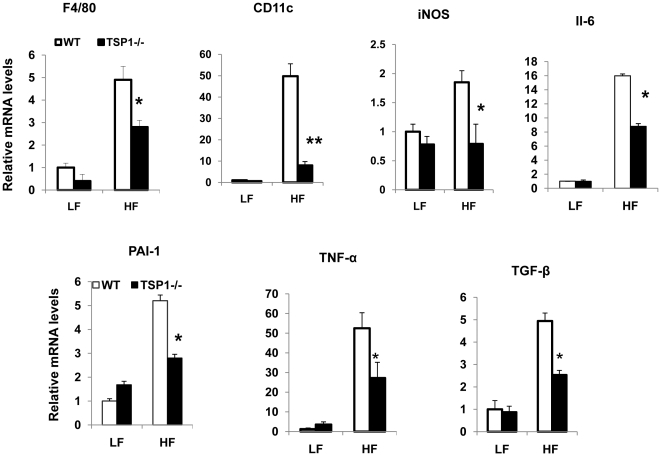
Obesity-induced inflammation was reduced in adipose tissue from TSP1-/- mice. Relative mRNA expression of macrophage and inflammatory markers in epididymal fat tissue from WT or TSP1-/- mice fed a LF or HF diet was determined by real-time PCR. Data are represented as mean±SE (n = 3 mice/group). *P<0.05 vs. WT HF group. **p<0.01 vs. WT HF group.

**Figure 5:**
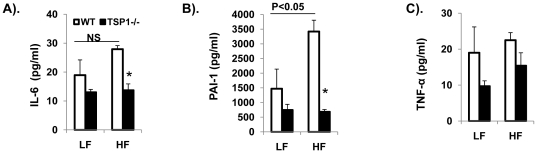
Obesity induced systemic inflammation was reduced in TSP1-/- mice. Plasma IL-6, TNF-α, and PAI-1 levels were measured as described in Material and Methods section. Data are represented as mean±SE (n = 6 mice/group). *P<0.05 vs. WT HF group.

Recent studies suggest that adipose tissue macrophages (ATMs) play a critical role in obesity associated chronic inflammation and insulin resistance [29]. Therefore, we determined the effect of TSP1 deficiency on ATM accumulation in adipose tissue using both immunohistochemical staining and real-time PCR. As shown in **Figure 3**, high fat feeding significantly increased F4/80 positive macrophage accumulation and crown like structure (CLS) in adipose tissue of wild type mice. However, ATM accumulation was increased to a lower extent in the HF-fed TSP1-/- mice. This immunohistochemical staining result was confirmed by real-time PCR showing that F4/80 mRNA levels were increased to a significantly lower extent in HF-fed TSP1-/- mice (**Figure 4**). We also observed this relationship in the expression of CD11c in the adipose tissue of two genotypes. Again, obese TSP1-/- mice had significantly lower CD11c levels in adipose tissue as compared to obese WT control (**Figure 4**). CD11c is a marker for a subset of proinflammatory immune cells that have been shown to play an important role in obesity-induced insulin resistance [30]. Other inflammatory cytokines such as iNOS, IL-6, TNF-α, PAI-1 and TGF-β were also reduced in the adipose tissue from the obese TSP1-/- mice (**Figure 4**). Furthermore, in WT mice, plasma PAI-1 levels were significantly increased and IL-6 levels had a trend in increase in HF-fed WT mice as compared to LF-fed WT mice. However, in TSP1-/- mice, neither plasma PAI-1 levels nor IL-6 levels were changed in HF-fed TSP1-/- mice as compared to LF-fed TSP1-/- mice. In addition, as compared to HF-Fed WT mice, both PAI-1 and IL-6 levels were significantly decreased in HF-fed TSP1-/- mice (**Figure 5**). Together, the data indicate that obese TSP1-/- mice have significantly decreased macrophage accumulation in adipose tissue and reduced systemic and local inflammatory cytokine levels.

### Macrophages from TSP1-/- mice have a reduced inflammatory phenotype and migratory ability

**Figure 6:**
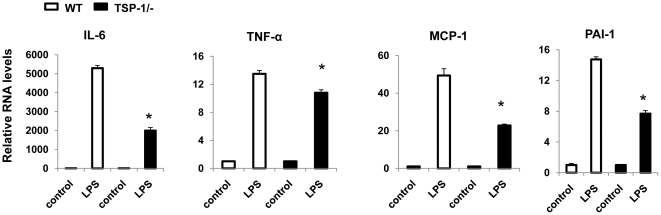
Macrophages from TSP1-/- mice had a reduced inflammatory phenotype. Bone marrow derived macrophages (BMDM) from WT or TSP1-/- mice were treated with or without LPS (100 ng/ml) for 3 hr. After treatment, the cells were harvested and relative gene expression of inflammatory cytokines was determined by real-time PCR. Data are represented as mean±SE (n = 3 experiments). *P<0.05 vs. WT LPS group.

**Figure 7:**
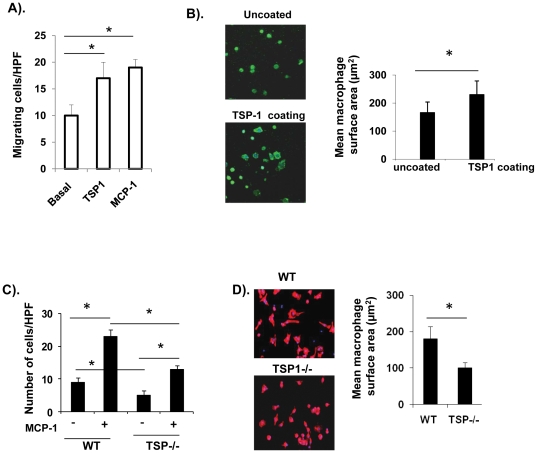
Effect of TSP1 on macrophage migration and adhesion. Peritoneal macrophages were isolated from WT or TSP1-/- mice as described in Materials and Methods Section. (A). Ability of WT macrophages to migrate toward purified TSP1 (5 µg/ml) or MCP-1 (50 ng/ml) using modified Boyden Microchemotaxis Chamber. (B). Ability of WT macrophages to spread on LAB-TEK slides precoated with purified TSP1 (5 µg/ml) was determined as described in Materials and Methods section. Cell surface area was quantified. (C). Migration of WT or TSP1-/- macrophages toward MCP-1 (50 ng/ml) was determined. (D). Spreading of WT or TSP1-/- macrophages on fibronectin coated slides was determined and cell surface area was quantified. The graph depicts the mean±SE of three separate experiments. * p<0.05.

**Figure 8:**
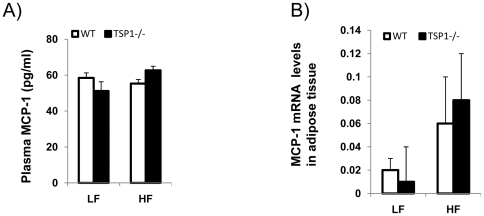
MCP-1 levels in plasma and in adipose tissue from four groups of mice. (A). Plasma MCP-1 levels were measured using a mouse adipokine assay kit (from Millipore); (B). MCP-1 mRNA levels in adipose tissue from four groups of mice were determined by real-time PCR. Data ate represented as mean±SE (n = 3 mice/group).

**Figure 9:**
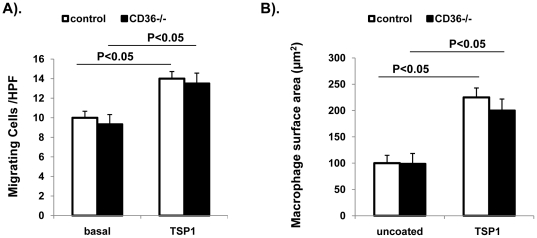
Effect of TSP1 on macrophage migration and adhesion from WT and CD36 -/- mice. Peritoneal macrophages were isolated from WT or CD36 -/- mice. (A). Ability of macrophages to migrate toward purified TSP1 (5 µg/ml) was determined using modified Boyden Chamber. (B). Ability of macrophages to spread on LAB-TEK slides precoated with purified TSP1 (5 µg/ml) was determined. Data ate represented as mean±SE of three experiments.

Obesity is associated with increased systemic concentrations of fatty acids and endotoxin (lipopolysaccharide) that are able to induce an inflammatory response [31]. To further determine the role of TSP1 in macrophage function, bone marrow derived cells were isolated from wild type and TSP1-/- mice. These cells were differentiated into macrophages and treated with lipopolysaccharide (LPS) for 3 hr. Inflammatory cytokine gene expression was determined by real-time PCR. There was a significant decrease in the gene expression of IL-6, TNF-α, MCP-1 and PAI-1 in LPS treated macrophages from TSP1-/- mice (**Figure 6**). This suggests that the macrophages from TSP1-/- mice had a reduced inflammatory phenotype. We also determined the effect of TSP1 on macrophage migration and adhesion. As shown in **Figure 7A and B**, addition of purified TSP1 significantly increased migration and adhesion of wild type macrophage cells. Macrophages from TSP1-/- mice showed decreased migration and adhesion ability compared to WT macrophages (**Figure 7** **C and D**). The effect of TSP1 on macrophage migration might be MCP-1 independent since we did not find difference of MCP1 levels either in plasma or in adipose tissue between WT and TSP1-/- mice (**Figure 8**). In addition, CD36, a receptor of TSP1, may not be involved in TSP1 mediated macrophage migration (**Figure 9**). Together, the data suggests that TSP1 is an important regulator of macrophage function.

## Discussion

TSP1 is a multifunctional matricellular protein that is up-regulated in inflamed adipose tissue of obese mice and humans [26], [27], [32]. Previous studies suggest that TSP1 plays a role in obesity and insulin resistance [27]. In the present study, we examined the effect of TSP1 deficiency on the development of obesity and insulin resistance in a high fat diet induced obese mouse model. Using this diet-induced obesity paradigm, we first demonstrate that TSP1 deletion reduces inflammation and improves whole body insulin sensitivity in the obese state. The improved glucose-insulin homeostasis is associated with significantly decreased macrophage accumulation in adipose tissue and decreased adipose inflammation. In vitro studies further support the effect of TSP1 on macrophage mobility and function. Together, these data demonstrate that TSP1 is a key regulator of macrophage function and influences the inflammatory state, contributing to obesity-associated insulin resistance.

Our current study demonstrates that TSP1 deficiency does not affect the development of high fat diet induced obesity, which is in agreement with the report from Voros et al [32]. However, in contrast to their study, we found that the obese TSP1-deficient mice have significantly improved glucose tolerance and insulin sensitivity as compared to obese wild type control mice. This discrepancy may be due to several factors including differences in the length of feeding, age of mice, high fat diet composition, and methods to measure glucose tolerance and insulin sensitivity. In our study, we fed eight week old male TSP1deficient mice and wild type littermates with low fat diet (LF, 10% kcal as fat; D12450B; Research Diets, Inc, NJ) and a high fat diet from Research Diet (HF, 60% kcal as fat; D12492, Research Diet, Inc, NJ) for 16 weeks; whereas Voros et al fed five week old male TSP1 deficient mice or wild type mice with a high fat diet from Harlan (TD 88137, containing 42% Kcal as fat) for 15 weeks. In addition, we performed glucose tolerance and insulin sensitivity tests on animals after 6 hr fasting followed by an intraperitoneal injection of glucose (1 mg/g body weight) or insulin (0.5 unit/kg body weight); whereas Voros et al performed these tests in mice after overnight fasting followed by an intraperitoneal injection of glucose at 3 mg/g body weight. A recent report from Andrikopoulos et al indicated that different fasting periods and varying concentrations of glucose injections can dramatically affect the results of glucose tolerance test in mice [33]. Moreover, they demonstrated that blood glucose concentrations after 6 hr fasting are a better representation of blood glucose levels throughout the day. Therefore, varying fasting times and/or glucose concentrations may explain the difference between our study and Voros' report.

One important finding of our study is that the obese TSP1 deficient mice have improved glucose tolerance and insulin sensitivity. Importantly, improvement in glucose-insulin homeostasis in obese TSP1 deficient mice was observed even though mice exhibited similar levels of obesity as wild type controls. Moreover, our results suggest that the improved metabolic profile of TSP1 deficient mice is partially due to the effect of TSP1 gene deletion on inflammation. We found that systemic and adipose tissue inflammation is significantly reduced in obese TSP1 deficient mice compared to obese wild type controls. This is associated with decreased accumulation of macrophages in fat tissue. Rodent and human studies suggest that adipose tissue macrophages play a critical role in obesity associated chronic inflammation and insulin resistance [29]. Other studies found that obesity is strongly associated with the accumulation of proinflammatory macrophages (F4/80^+^ cells) that express CD11 c (a dendritic cell marker) in adipose tissue [4]. These F4/80 + CD11c+ cells are bone marrow derived adipose tissue macrophages that selectively localize to the crown like structure surrounding dead adipocytes. These cells play an important role in obesity associated metabolic profiles [30], [34]. Our data suggest an important role of TSP1 in regulating CD11c + macrophage infiltration and inflammation based on the following observations: 1) Immunohistochemical results showed that the frequency of the crown like structure and F4/80 + macrophages were dramatically decreased in adipose tissue of obese TSP1 deficient mice; 2) Gene expression of F4/80 and CD11c were significantly decreased in adipose tissue of obese TSP1 deficient mice; 3) mRNA levels of proinflammatory cytokines such as IL-6, TNF-α, and PAI-1 were significantly decreased in adipose tissue of obese TSP1 deficient mice; 4) Bone marrow derived macrophages from TSP1 -/- mice exhibited a reduced inflammatory phenotype.

Furthermore, our in vitro data demonstrate that TSP1 stimulates macrophage migration and adhesion. This result is in agreement with previous studies showing that TSP1 can act as a monocyte chemoattractant [20], [21]. Previous studies have shown that TSP1 did not stimulate MCP-1 release from differentiated U937 human monocytic cells. However, they found that PAI-1 levels in monocytes or murine macrophages were significantly increased by TSP1 [21], suggesting that the effect of TSP1 on macrophage migration might be MCP-1 independent but PAI-1 dependent. Consistently, we did not find a difference of MCP1 levels in either plasma or adipose tissue between WT and TSP1-/- mice (figure 8). By using CD36 deficient macrophages, we demonstrate that CD36 (a receptor of TSP1) may not be involved in TSP1 mediated macrophage migration (figure 9). In addition to regulating macrophage migration, another study demonstrated that TSP1 deficient murine macrophages exhibit an increased capacity for FcγR-mediated phagocytosis [35]. Therefore, in an obese state, it is possible that TSP1-/- deficient macrophages could rapidly clear dead adipocytes contributing to decreased inflammation. TSP1 may also influence other immune cells such as T cells contributing to obesity-induced adipose tissue inflammation. Future studies will explore this possibility.

TSP1 is a major regulator for latent TGF-β activation in vitro as well as in vivo [36], [37], [38], [39], [40]. Studies have demonstrated that increased TGF-β activity and its downstream target PAI-1 are associated with obesity, inflammation and insulin resistance [41], [42], [43]. In this study, we found that TGF-β downstream molecular-PAI-1 levels in plasma and adipose tissue were significantly decreased in the obese TSP1-/- mice. This suggests that decreased TSP1 dependent TGF-β activity may contribute to the reduced systemic and local inflammation and improved insulin sensitivity that was observed in the obese TSP1-/- mice. An ongoing in vivo study is currently exploring this mechanism using an antagonist of TSP1-dependent TGF-β activation.

In summary, results from this study demonstrate an important role for TSP1 in regulation of macrophage function and in obesity-induced inflammation and insulin resistance. TSP1 depletion in an obese state prevents the accumulation of macrophages in adipose tissue and pro-inflammatory cytokine expression in peripheral tissues, resulting in improved insulin sensitivity. A direct effect of TSP1 on macrophage motility and function was also demonstrated in our current studies. The results of this study together with the report of increased TSP1 in human obesity [27] suggest that TSP1 may be a potential target of the inflammatory and metabolic complications of obesity.
